# Coordination of Flower Maturation by a Regulatory Circuit of Three MicroRNAs

**DOI:** 10.1371/journal.pgen.1003374

**Published:** 2013-03-28

**Authors:** Ignacio Rubio-Somoza, Detlef Weigel

**Affiliations:** Department of Molecular Biology, Max Planck Institute for Developmental Biology, Tübingen, Germany; Peking University, China

## Abstract

The development of multicellular organisms relies on interconnected genetic programs that control progression through their life cycle. MicroRNAs (miRNAs) and transcription factors (TFs) play key roles in such regulatory circuits. Here, we describe how three evolutionary conserved miRNA-TF pairs interact to form multiple checkpoints during reproductive development of *Arabidopsis thaliana*. Genetic, cellular, and physiological experiments show that miR159- and miR319-regulated MYB and TCP transcription factors pattern the expression of miR167 family members and their ARF6/8 targets. Coordinated action of these miRNA-TF pairs is crucial for the execution of consecutive hormone-dependent transitions during flower maturation. Cross-regulation includes both cis- and trans-regulatory interactions between these miRNAs and their targets. Our observations reveal how different miRNA-TF pairs can be organized into modules that coordinate successive steps in the plant life cycle.

## Author Summary

Development of multicellular organisms relies on properly timed execution of different genetic programs. An example is provided by developmental progression of flowers, which begins with the initiation of individual organs, followed by differentiation, growth, and finally production of the gametes. This article investigates the contribution of three microRNAs (miRNAs) and the transcription factors (TFs) that are regulated by these miRNAs to this process. Two of the miRNA-TF pairs act early to control in parallel the activity of the third miRNA-TF pair, which in turn modulates hormone programs that drive organ maturation and reproduction. Importantly, the two upstream TFs directly interact to regulate expression of the downstream miRNA. The results described here demonstrate how miRNA-TF pairs can be organized into regulatory circuits, with independent miRNA-TF pairs converging on common downstream genes.

## Introduction

In mammals, reproductive organs are already formed in the embryo, with gametogenesis being completed after birth. By contrast, plant reproductive development is initiated only after the transition from the juvenile to the adult phase, with the production of flowers that harbor the reproductive organs. Despite these differences, both kingdoms tightly control the progression from initiation to maturation of reproductive organs and the acquisition of reproductive competence. The progression relies on successive genetic programs that govern a delicate balance of cell division and differentiation, organ growth and maturation, as well as cell death. Coordination of these events in plants makes use of a host of interacting hormones, including cytokinins, auxin, gibberellins (GAs), jasmonate (JA), ethylene and brassinosteroids. Hormone action is often mediated by dedicated transcription factors (TFs) such as the auxin response factors (ARFs), several of which are microRNA (miRNA) regulated. As an example, expression of the paralogous master regulators *ARF6* and *ARF8* (“*ARF6/8*”) of *Arabidopsis thaliana* are fine-tuned in specific floral organs by miR167 [1]. Increased miR167 levels or reduced ARF6/8 function both result in underdeveloped floral organs and impaired male and female fertility [1]–[4]. *ARF6/8* activity is also sufficient for driving growth of floral organs, as shown with plants in which miR167 function is blocked [1], [5]. ARF6/8 regulate the expression of auxin homeostatic genes and help to set the boundaries of cytokinin-dependent meristematic activity, in addition to modulating JA biosynthesis [2], [4], [6]. Therefore, some of the defects in flowers with reduced *ARF6/8* levels resemble symptoms of auxin, GA and JA deficiency and cytokinin overproduction [7]–[14]. Further complexity comes from miR167 having several isoforms that differ in their expression patterns and in their ability to downregulate their ARF6/8 targets [1].

Defects seen in *arf6/8* mutant flowers are reminiscent of ones observed when the function of two other miRNAs, miR159 and miR319, is compromised. Such flowers suffer from retarded development of organs in the three outer whorls, the sepals, petals and anthers. The targets of these miRNAs are MYB and TCP transcription factors [15]–[18]. miR159-mediated restriction of MYB33 and MYB65 activity to anthers is necessary for normal floral organ growth and fertility [16], [19], [20]. MiR159 levels are positively regulated by GA [15], and at least in rice, this is also true for miR319 expression [21]. MiR319 is encoded by three genomic loci with overlapping expression patterns in *A. thaliana*. Transcription of *MIR319A* coincides with *MIR319C* at the base of all floral organs, in stamen filaments and petals, while *MIR319B* is restricted to stamens and the abscission zone of sepals [22], [23]. Reduction of miR319 activity results in smaller flowers with short petals, strap-like petals and underdeveloped stamens; in extreme cases, petals and stamens are lost [5], [23].

Here, we report how these three evolutionary conserved miRNA-TF pairs (miRNA-TF nodes hereafter) are organized into a regulatory network that is responsible for several checkpoints in floral organ maturation. MiR159 and miR319 regulate directly interacting targets, which in turn control the expression of *MIR167* family members during this process. Restriction of *MIR167A* expression allows the progression from meristematic, cytokinin-dependent programs to auxin-dependent organogenesis, culminating in GA- and JA-dependent maturation. We propose that the convergence of unrelated miRNA targets onto common downstream targets is an important feature of miRNA networks in plant development.

## Results

### Floral defects caused by altered miR159, miR167, and miR319 activities

**Figure 1:**
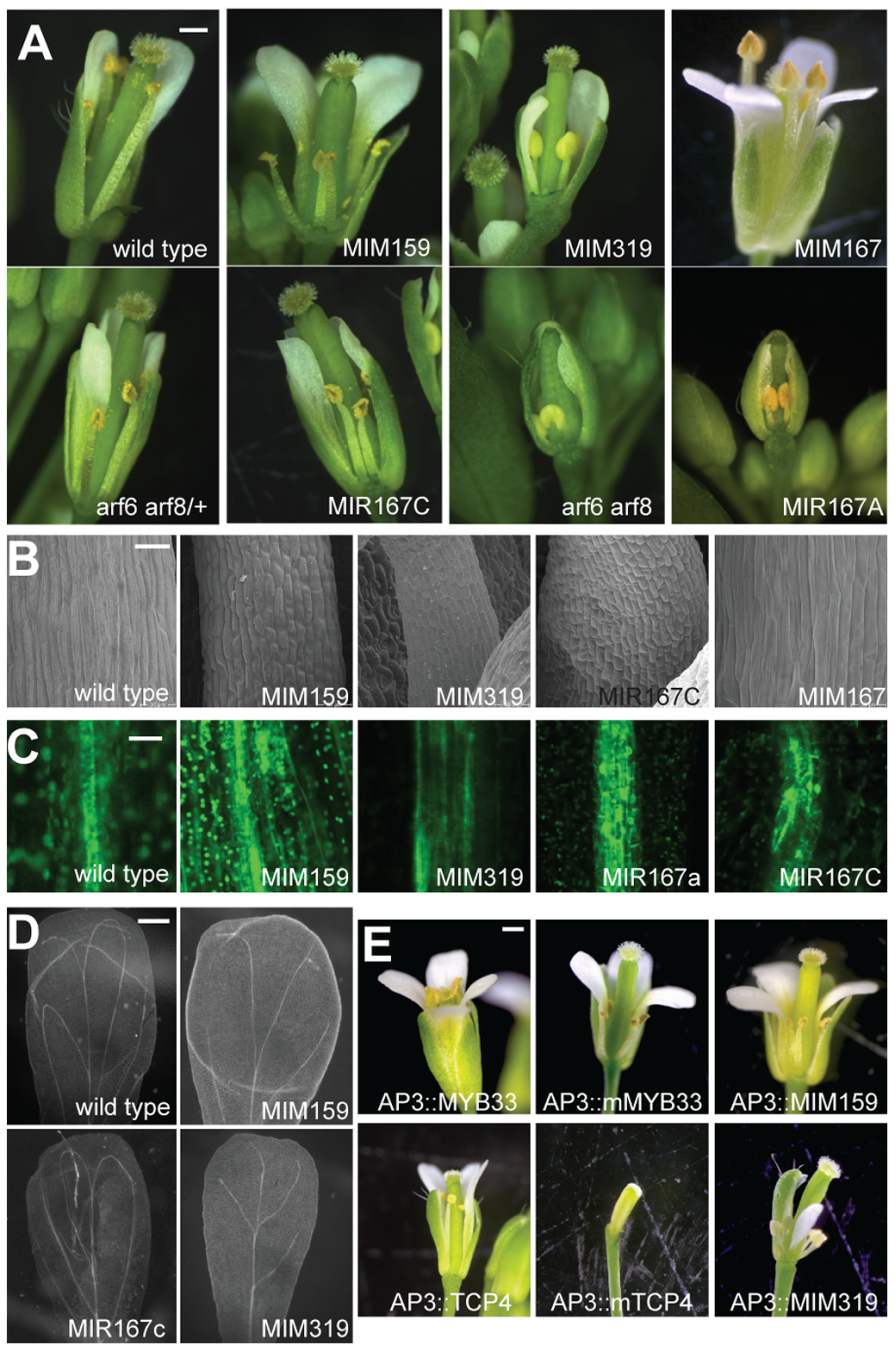
Effects of miR159, miR167, and miR319 on flower morphology. (A) Entire flowers, with some petals and sepals removed to reveal interior organs. Petals and stamens are underdeveloped in *Pro35S:MIM159* (‘MIM159’), *Pro35S*:*MIM319* (‘MIM319’), *Pro35S:MIR167A* (‘MIR167A’), *Pro35S:MIR167C* (‘MIR167C‘) plants and *arf6 arf8* mutants, all of which have reduced ARF6/8 activity. *Pro35S:MIM167* (‘MIM167’) plants, which have increased ARF6/8 activity, show an opposite phenotype of organ overgrowth, especially in anther filaments. (B) Epidermis of stamen filaments. (C) Reduced ARF6/8 activity in different transgenic backgrounds leads to expanded expression of procambial marker Q0990. (D) Effects of reduced ARF6/8 activity on vasculature pattern in mature petals (dark field view of cleared organs). (E) Entire flowers of plants in which miR159 and miR319 targets are specifically overexpressed or their functions attenuated in petals and stamens. Scale bars indicate 1 mm (A, E), 30 µm (B), 10 µm (C), 200 µm (D). See also Figure S2.

Flowers of *Pro35S:MIM159* and *Pro35S:MIM319* transgenic plants, in which miR159 and miR319 activities are impaired by constitutive expression of target mimics (Figure S1), have defects reminiscent of those found in plants with reduced ARF6/8 activity [5]. We therefore compared in detail the consequences of reducing ARF6/8, miR159 and miR319 function during reproductive development. Similar to plants with reduced ARF6/8 function (*arf6*-2 *arf8*-3 double mutants, *Pro35S:MIR167A* and *Pro35S:MIR167C* plants), the maturation of sepals, petals and anthers was delayed to various degrees in *Pro35S:MIM159* and *Pro35S:MIM319* plants. In addition, the flower stems, or pedicels, were shorter, and the angles of flowers relative to the main stem were more acute (Figure 1A and Figure S2). Stamens did not completely elongate in any of the genotypes. The strongest defects were seen in *arf6 arf8*, *Pro35S:MIR167A* and *Pro35S:MIM319* plants, which never shed pollen (Figure 1A). Thus, stamen filament elongation seemed to be more sensitive to loss of ARF6/8 activity than anther dehiscence.

Smaller organ size was likely due to reduced cell size, as seen by scanning electron microscopy of stamen filaments (Figure 1B). In addition to epidermal defects, vascular development in stamen filaments appeared to be arrested at the procambium stage, since the expression of the procambial marker Q0990 [24] was expanded in plants with diminished miR159, miR319 and ARF6/8 activities (Figure 1C). Petal vasculature was also compromised, lacking secondary loops in *Pro35S*:*MIM319* plants, or having only two instead of four loops in *Pro35S*:*MIM159* and *Pro35S:MIR167c* plants (Figure 1D).

Since petals and stamens were particularly sensitive to depletion of miR159 and miR319, we investigated the specific functions of two of their main targets, *MYB33* and *TCP4* [16], [17], [19], [20], [25], by expressing miRNA-non-targetable versions (*mMYB33* and *mTCP4*) under the control of the petal- and stamen-specific *APETALA3* (*AP3*) promoter [26]. We grew those plants along with plants expressing *MIM159* and *MIM319* decoys under the same *AP3* promoter for comparison (Figure 1E). While mis-expression of the unmodified versions had little, if any, phenotypic effects, the non-targetable forms led to underdeveloped petals and anthers in *ProAP3*:*mMYB33* flowers, and elimination of petals and anthers in *ProAP3*:*mTCP4* plants (Figure 1E) [23]. Flowers expressing the miRNA insensitive form of *MYB33* faithfully resembled the developmental impairments found in petals and stamens of *ProAP3:MIM159* plants (Figure 1E). Nevertheless, flowers from *ProAP3:mTCP4* presented more severe developmental defects than *ProAP3:MIM319* flowers, in line with previous reports [23]. The phenotypic differences might be explained by a crosstalk between different miR319 TCP targets, or, as suggested in [23], by TCP4 movement or feed-forward regulation.

### A sequence of hormone-related events regulated by coordinated miRNA action

Previous studies that have examined the role of different hormones during flower maturation suggested a sequential pattern for hormone action in the three outer whorls of the flower, especially in petals and stamens. Early in flower development, cytokinin-dependent maintenance of meristematic activity is delimited by the action of auxins [2]. Auxins subsequently trigger GA- and JA-based programs required for organ elongation and maturation during floral development [4], [27], [28].

**Figure 2:**
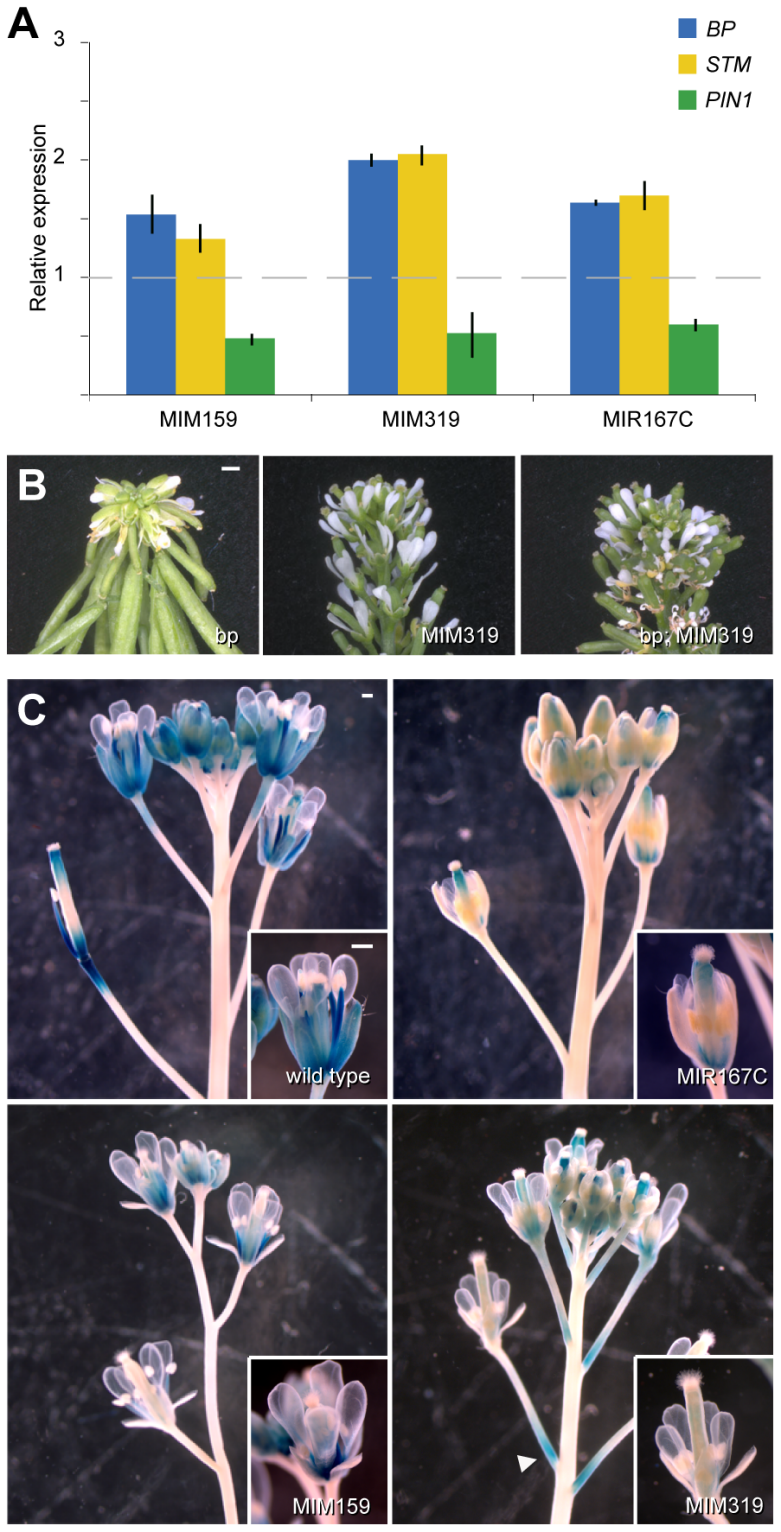
Effects of miRNA mis-regulation on hormone pathways important for flower formation and maturation. (A) Expression of genes involved in cytokinin-signaling (*BP* and *STM*) and auxin transport (*PIN1*) in flowers, as monitored by real-time RT-PCR. Error bars indicate range of two biological and two technical replicates. Measurements in mutants were normalized to values from wild-type inflorescences (dashed line). (B) *Pro35S:MIM319* suppresses the downward-bent pedicels in *bp*-1 mutants (L*er* background). (C) Misregulation of miR159, miR167 or miR319 reduces *ProLOX2*:*GUS* reporter activity, which is most active in sepals and stamens of wild-type flowers. Arrowhead points to ectopic GUS activity at the base of *Pro35S:MIM319* pedicels. Scale bars indicate 1 mm.

A major consequence of *ARF6/8* deficiency, and therefore reduced auxin signaling, is the expanded expression of positive meristem regulators involved in cytokinin action, such as the class I *KNOX* genes *SHOOTMERISTEMLES*S (*STM*) and *BREVIPEDICELLUS* (*BP*) [2]. The phenotypic alterations described above, along with premature flower bud opening, might thus be attributed to the expansion of cytokinin-related meristematic activity and reduced auxin action [12], [13], [29], [30]. As similar developmental defects were found in plants deficient in miR159 and miR319 activity, we asked whether their absence was also affecting the sequence of hormone-related events leading to flower maturation. We analyzed *STM* and *BP* activity in *Pro35S*:*MIM159* and *Pro35S:MIM319* inflorescences, and in *MIR167C* overexpressors, which have reduced ARF6/8 expression. In all three backgrounds, *STM* and *BP* expression were increased (Figure 2A). Suppression of the *bp*-1 mutant phenotype by *Pro35S:MIM319* indicated that increased expression of other *KNOXI* genes could bypass the *BP* requirement (Figure 2B, 2C). Increased *KNOXI* activity might also explain the abnormal angle of petiole growth in mutants affected in ARF6/8, miR159 and miR319 activities. Additional defects caused by increased *KNOXI* activity are in vasculature development and stamen elongation and maturation, which are linked to reduced auxin transport [2], [10]. Accordingly, *STM* and *BP* upregulation was paralleled by reduced expression of the *PIN1* auxin transporter gene in *Pro35S:MIM159*, *Pro35S:MIR319* and *Pro35S:MIR167C* lines (Figure 2A). These results showed that miR159, miR319, just like ARF6/8, were negative regulators of the expression of class I *KNOX* genes, thereby confining cytokinin-dependent meristematic activity.

In addition to demarcating cytokinin action, ARF6/8 also contribute to flower development in later stages of maturation. ARF6/8 activate the expression of genes encoding two major JA biosynthetic enzymes, *LOX2* and *DAD1* [2], [4], a role that they share with the miR319 target TCP4 [31]. JA is important for the latest stages of floral organ development, including final stamen elongation, positioning of the anthers at the stigma at the time of dehiscence, and pollen maturation. Hence, without JA, plants are infertile [32]–[34]. In addition, JA modulates petal growth and vascular development [35], [36]. *LOX2* is not essential for fertility [37], but the *LOX2* promoter is active in stamens and sepals, where its expression increases from stages 12 to 15 of flower development [38], [39]. Consistent with a role of miR159, miR167 and miR319 in JA regulation, *LOX2* promoter activity was reduced in sepals of *Pro35S:MIM319*, *Pro35S:MIM159* and *Pro35S:MIR167c* plants (Figure 2C), with ectopic activation at the base of *Pro35S*:*MIM319* pedicels, where *MIR319B* is normally expressed (Figure 2C, Figure S4C).

Taken together, the phenotypic and physiological resemblance of plants with a reduction in *ARF6/8* or miR159/miR319 activities supports links between miR167, miR159 and miR319 in growth and hormone-dependent maturation of sepals, petals and anthers. The coordinated roles of the three miRNAs and their targets initially enable organogenesis by confining cytokinin-dependent meristematic activity. Later on, they contribute to the JA-dependent maturation of floral organs.

### Mediation of miR159 and miR319 effects by miR167

**Figure 3:**
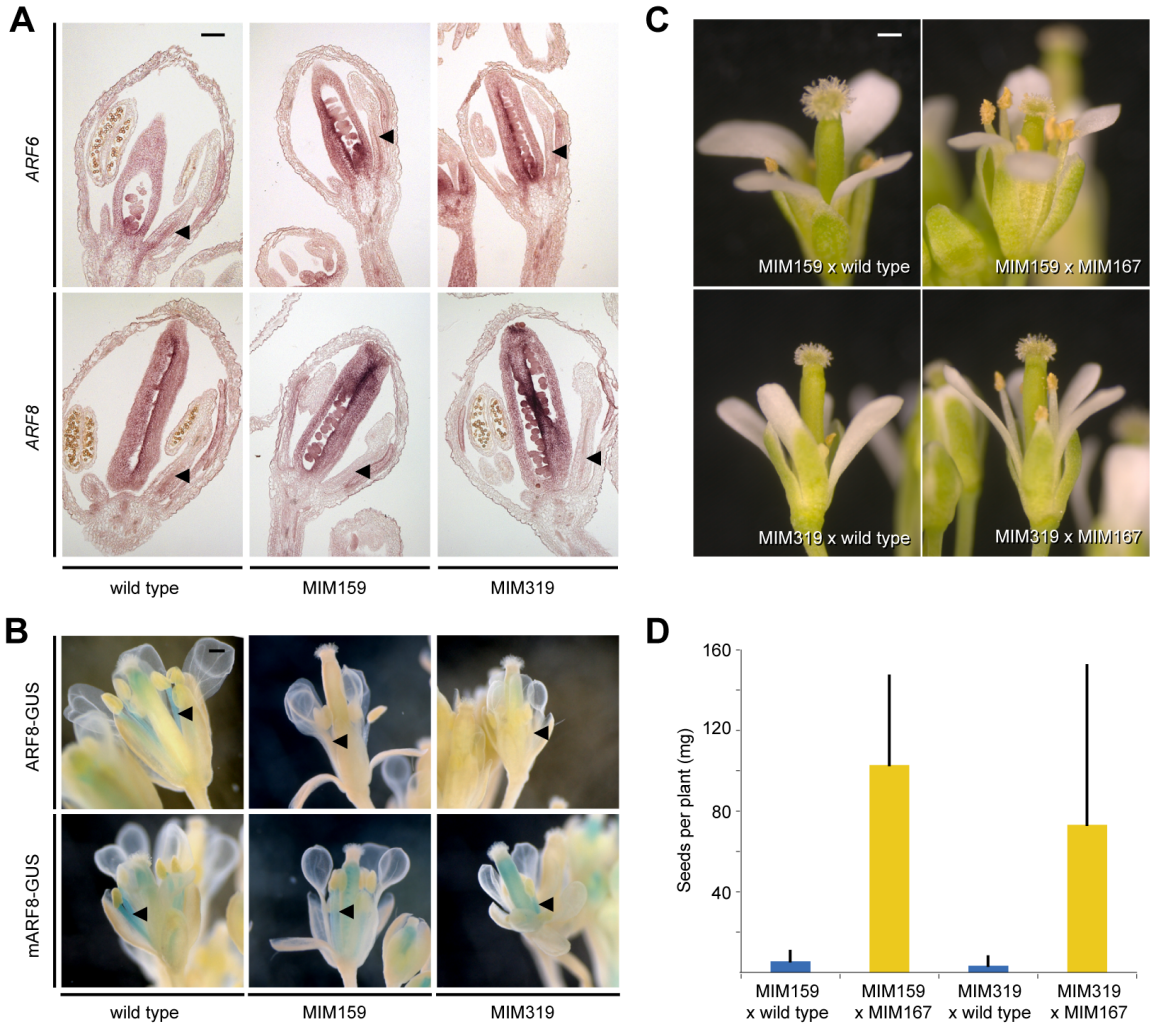
miR167-dependent regulation of ARF6/8 by miR159 and miR319. (A) *ARF6/8* RNA expression in stamen filaments (triangles). (B) *ARF8* reporter activity in stamen filaments (triangles). (C) Partial suppression of floral organ defects triggered by Pro35S:*MIM159* and *Pro35S:MIM319* upon reduction of miR167 activity. (D) Suppression of fertility defects caused by *Pro35S:MIM159* (p<<0.001) and Pro35S:*MIM319* (p<0.001). Error bars indicate standard deviation for at least 10 individuals from two independent crosses. Scale bars indicate 100 µm (A), 1 mm (B, C).

We next assayed *ARF6/8* expression in *Pro35S:MIM159* and *Pro35S:MIM319* plants in order to determine how *ARF6/8* are regulated by miR159 and miR319. In wild type, both genes are expressed in the vasculature of floral organs, including in sepals, in petals, in stamen filaments, and in the precursor of the transmitting tract, the medial ridge of the carpel [1]. Expression of *ARF6/8* was slightly reduced in *Pro35S:MIM159* and *Pro35S:MIM319* flowers, especially in the stamen filament vasculature (Figure 3A). In addition, we found that the expression of the *ProARF8:ARF8-GUS* reporter was specifically absent from stamens when miR159 and miR319 activities were reduced (Figure 3B).

To determine whether miR159 and miR319 targets are likely to regulate *ARF6/8* directly or through miR167, we compared the response of *ProARF8*:*ARF8-GUS* and its miRNA insensitive form, *ProARF8*:*mARF8-GUS*, to changes in miR159 and miR319 activity (Figure 3B). While the expression of *ProARF8:ARF8-GUS* was altered in *Pro35S:MIM159* and *Pro35S:MIM319* plants, that of *ProARF8*:*mARF8-GUS* was not, indicating that miR159 and miR319 targets regulate *ARF8* by increasing miR167 activity (Figure 3B). This hypothesis was further supported by the finding that petal and stamen development as well as fertility in *Pro35S:MIM159* and *Pro35S:MIM319* flowers were substantially restored when we reduced miR167 activity with the *Pro35S:MIM167* construct (Figure 3C, 3D). These results placed miR159 and miR319 upstream of miR167. Plant miRNAs can regulate the expression of their targets through mRNA cleavage and/or translational inhibition [5], [40]. The observed difference between the effects of changes in miR167 expression on *ARF6/8* mRNA and protein indicated that miR167 acts through both mRNA cleavage and translational inhibition.

**Figure 4:**
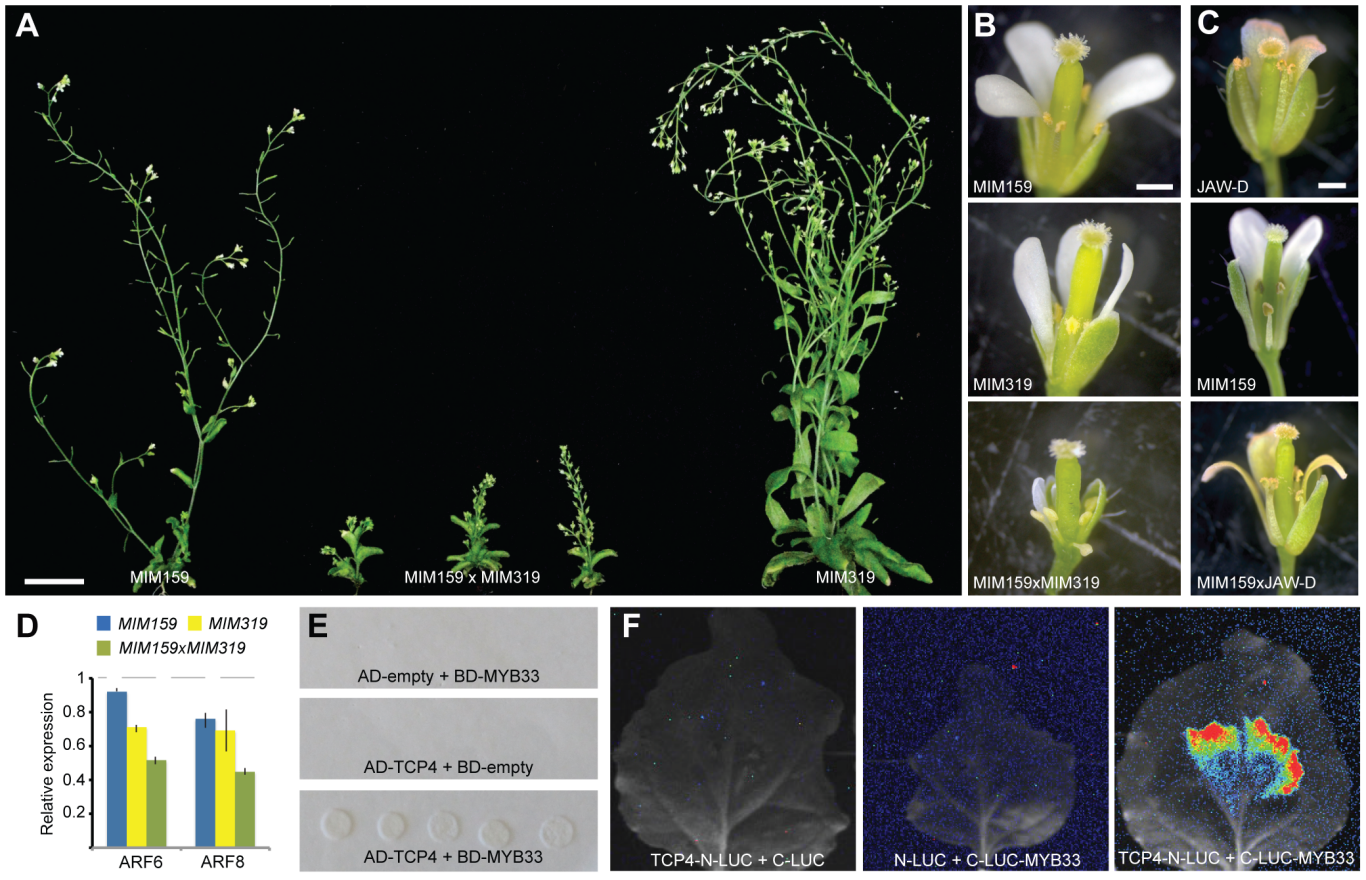
Interaction of miR159 and miR319 targets. (A) Comparison of 45-day old plants. (B) Close-up of flowers in single and double *Pro35S:MIM159* and *Pro35S:MIM319* expressing plants. (C) Altered floral development as consequence of reduced miR159 activity and/or miR319 overexpression. (D) *ARF6/8* expression levels in single and double *Pro35S:MIM159* and Pro35S:*MIM319* plants as monitored by real-time RT-PCR. Error bars indicate range of two biological and two technical replicates. Measurements in mutants were normalized to values from wild-type inflorescences (dashed lines). (E) Interaction of miR159 target MYB33 and miR319 target TCP4 in yeast-two-hybrid system. (F) Interaction of MYB33 and TCP4 assayed by firefly luciferase complementation in *N. benthamiana*. Luciferase activity is shown in false color, with highest levels red and lowest levels blue. Scale bars indicate 1 mm (B,C).

Simultaneous sequestration of both miR159 and miR319 resulted in additive phenotypic and molecular effects (Figure 4A, 4B, 4D), suggesting that the miR159- and miR319-regulated MYB and TCP transcription factors regulate shared target genes. We found that miR159 targets affected anther and petal development even when miR319 targets were suppressed in *jaw*-D plants (Figure 4C), indicating that MYBs and TCPs work in parallel to trigger miR167-mediated ARF6/8 regulation. Since TFs that regulate the same gene often form higher-order heteromeric complexes [41], we hypothesized that this might be the case for miR159-targetd MYBs and miR319-targeted TCPs. Consistent with this idea, the expression patterns of *MYB33* and *TCP4* in the affected floral organs overlap (Figure S3) and the proteins can interact in yeast and plant assays (Figure 4E, 4F). This implies that the two miRNA pathways converge on common downstream targets by modulating the composition of regulatory TF complexes.

### Differential regulation of *MIR167* genes by miR159 and miR319 targets

The divergent promoter activities of *MIR167* family members are paralleled by their different abilities to downregulate ARF6/8, both of which indicate subfunctionalization [1]. It appears likely that the different *MIRNA167* genes fine-tune ARF6/8 expression within different organs. To better understand miR159- and miR319-dependent regulation of *MIR167* genes, we assayed the transcriptional activity of *MIR167A*, *MIR167B* and *MIR167C* in *Pro35S:MIM159* and *Pro35S:MIM319* plants. We also analyzed *Pro35S:MIR167C* plants deficient in ARF6/8 function, to address whether altered reporter expression was a direct consequence of the absence of miR159 and miR319, or a downstream consequence of disrupting development by reducing ARF6/8 levels.

**Figure 5:**
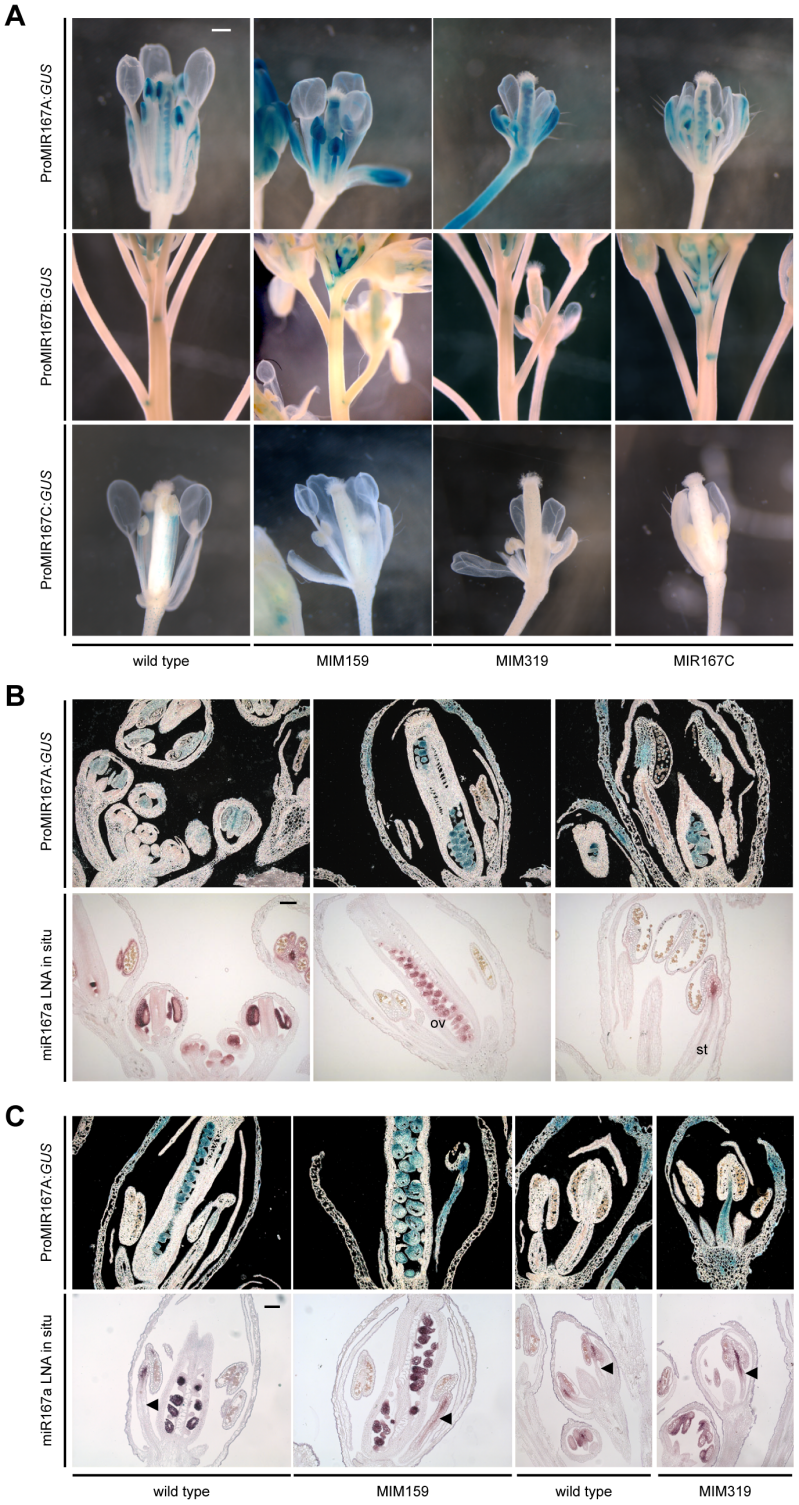
miR159- and miR319-dependent expression of miR167 in inflorescences. (A) Promoter activity of *MIR167* family members. Some sepals and petals have been removed to reveal interior organs. (B) Comparison of *MIR167A* promoter activity (darkfield images of sections, top rows) and mature miR167a expression in wild-type flowers. ov, ovule; st, stamen. (C) Comparison of *MIR167A* promoter activity and mature miR167a expression in transgenic plants, in which miR167a becomes ectopically activated in stamen filaments (triangles). Scale bars indicate 1 mm (A), 100 µm (B, C). See also Figure S4.

A GUS reporter under control of the *MIR167A* promoter is expressed in anther procambium, sepal vasculature and ovaries; activity of the *MIR167B* promoter has been detected in ovaries and at the base of flower pedicels; and the *MIR167C* promoter is mainly active in stamen filaments and petals [1]. As expected, in *Pro35S:MIM159* and *Pro35S:MIM319* flowers, *ProMIR167A:GUS* was ectopically expressed in tissues with abnormal development, including sepal vasculature, petals and stamen filaments, while *MIR167C* overexpression had no effect (Figure 5A and Figure S4A, S4B). In addition, similarly to the *LOX2* promoter in *Pro35S:MIM319* flowers (Figure 2C), the *MIR167A* promoter was ectopically activated at the base of pedicels, in contrast to the repression of the *MIR167B* promoter in this region (Figure 5A and Figure S4A, S4C). Likewise, reduced ARF6/8 function in *Pro35S:MIM159* and *Pro35S:MIM319* plants or in *MIR167C* overexpressors caused the *MIR167C* promoter to be less active in stamen filaments (Figure 5A). These results point to miR159 and miR319 as coordinating the expression pattern of *MIR167* family members in petals, sepals and stamen.

**Figure 6:**
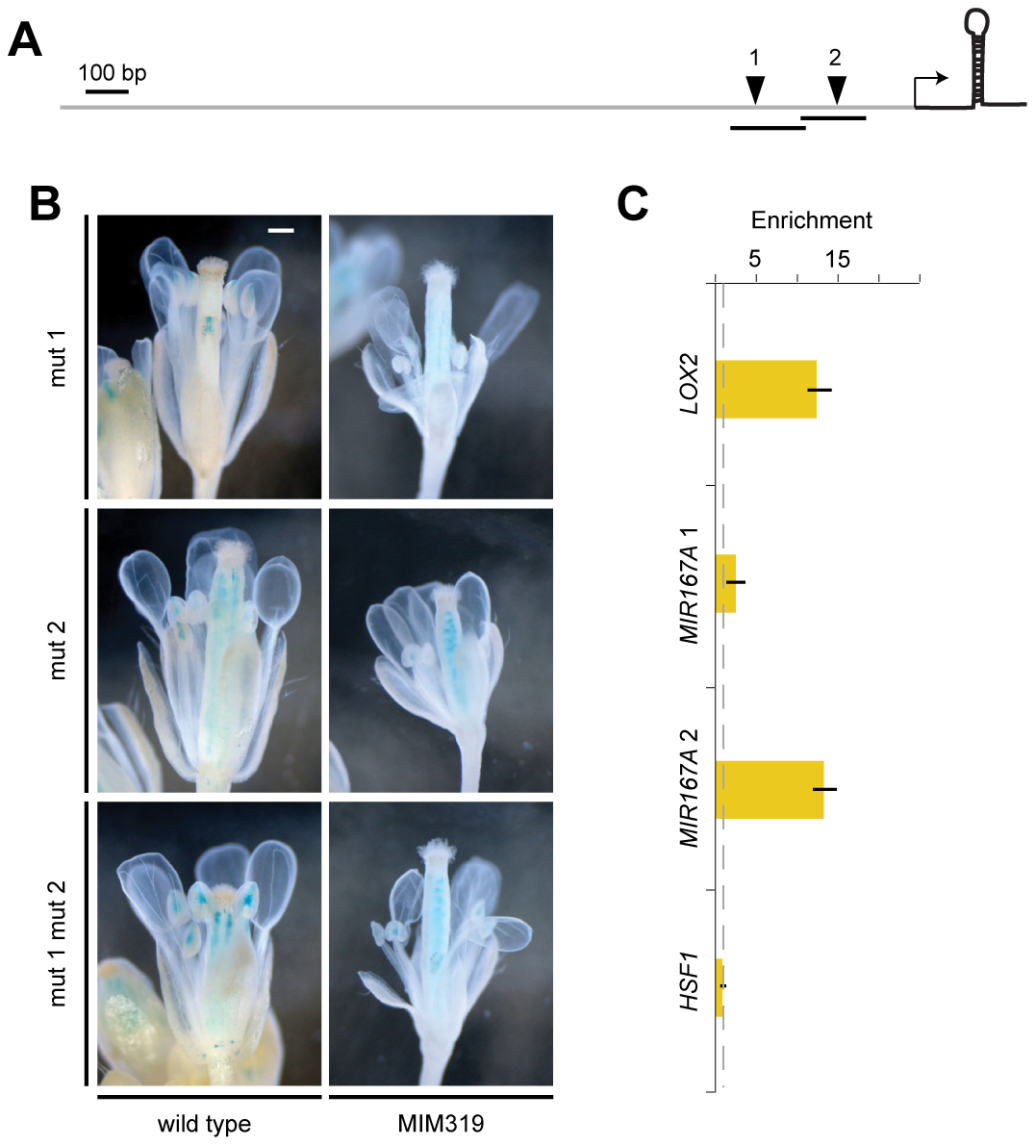
Direct regulation of *MIR167A* by TCP4. (A) Diagram of *MIR167A* promoter with potential TCP binding sites (inverted triangles) that were mutated (1: 5′-G[G/t]TCCC-3′; 2: 5′-GGA[C/a]CA-3′; lower case indicates mutant). Transcription start site is from [70]; transcribed region is not to scale. (B) Reporter gene assay with *ProMIR167A:GUS*. In contrast to the wild-type promoter (Figure 4A), the mutant promoter variants do not respond to a change in miR319 activity. Scale bar indicates 1 mm. (C) TCP4-GFP ChIP with material from *ProTCP4:TCP4*-GFP *Pro35S:MIM319* inflorescences, normalized against empty vector control (dotted line). Amplicons 1 and 2 are indicated as lines in (A); *LOX2* served as positive, *HSF1* as negative control. Error bars indicate range of two biological and two technical replicates. See also Tables S1, S2 and Figure S5.

*MIR167A* promoter activity correlated with mature miRNA levels, as shown by *in situ* hybridization with an LNA probe (Figure 5B). *MIR167A* was initially expressed in emerging stamen, becoming confined to the procambium of stamen filaments from stage 7 on, and its levels were increased in *Pro35S:MIM159* and *Pro35S:MIM319* flowers (Figure 5C). The expression pattern of miR167a in ovaries, which was unaffected by changes in miR159 and miR319, resembled its promoter activity as well (Figure 5B, 5C) [1], [42]. Together, these results suggested that the effects of miR159 and miR319 are mainly mediated by *MIR167A*. The *MIR167A* promoter has two predicted TCP binding sites (Table S1). Mutating either of two potential TCP sites rendered the *ProMIR167A:GUS* reporter unresponsive to increased TCP levels in *Pro35S:MIM319* plants (Figure 6A, 6B, and Figure S5A). We tested whether *MIR167A* regulation by TCP4 is direct by chromatin immunoprecipitation (ChIP) using anti-GFP antibodies and extracts from *ProTCP4:TCP4-GFP* Pro35S:*MIM31*9 inflorescences. Quantitative PCR revealed specific enrichment overlapping one of the two potential TCP binding motifs (Figure 6C, Table S1). The *A. thaliana* genome encodes 24 TCPs, including several in the miR319-regulated TCP4 clade, and many can homo- and heterodimerize (Figure S5B) [43]–[46]. It is therefore conceivable that one of the TCP consensus motifs is bound by a TCP other than TCP4.

## Discussion

We have analyzed how three miRNA-target nodes interact to control consecutive checkpoints during floral organ maturation in the three outer whorls. First, we have discovered that the miR159-MYB and miR319-TCP nodes can independently regulate the miR167-ARF node. Second, we conclude that the convergent downstream effects of miR159 and miR319 are at least partially due to direct interaction of their MYB and TCP transcription factor targets. The finding of a link between these three miRNA-TF nodes reinforces the observation that miRNAs in plants are disproportionately often involved in auxin signaling [47] and that they can be organized into miRNA networks [48].

The miR159-miR167-miR319 circuit acts in sepals, petals and anthers to modulate the activity of ARF6/8, which control a large number of floral genes [4]. MiR159 and miR319 dampen the expression of their TF targets, which can otherwise lead to miR167 misexpression both individually and cooperatively through engaging in common protein complexes. In addition to regulation by miR159 and miR319 targets, there is cross-regulation among *MIR167* genes, one example being repression of *MIR167C* in stamen filaments by miR167a. All these inputs are required to avoid miR167 misexpression. Moreover, the interactions can be complex, with miR319 contributing to activation of *MIR167B* and repression of *MIR167A* at the base of pedicels.

MiR159 and miR319 regulation enable ARF6/8 to play a central role in setting the cytokinin-auxin differentiation boundary by delimiting the expression of *KNOXI* genes. Inhibition of *KNOXI* expression leads to repression of cytokinin-based programs, which allows cells to exit the undifferentiated state [2], [12], [13]. MiR159- and miR319-dependent ARF6/8 function contributes to later aspects of development by promoting JA synthesis in sepals through the induction of *LOX2* expression and in petals and anthers through the regulation of *DAD1* expression [2]. In addition, *LOX2* is directly controlled by miR319-targeted TCP transcription factors at the base of pedicels independently of ARF6/8 levels. The tissue-specific differences in JA regulation by miR319 deserve further investigation.

**Figure 7:**
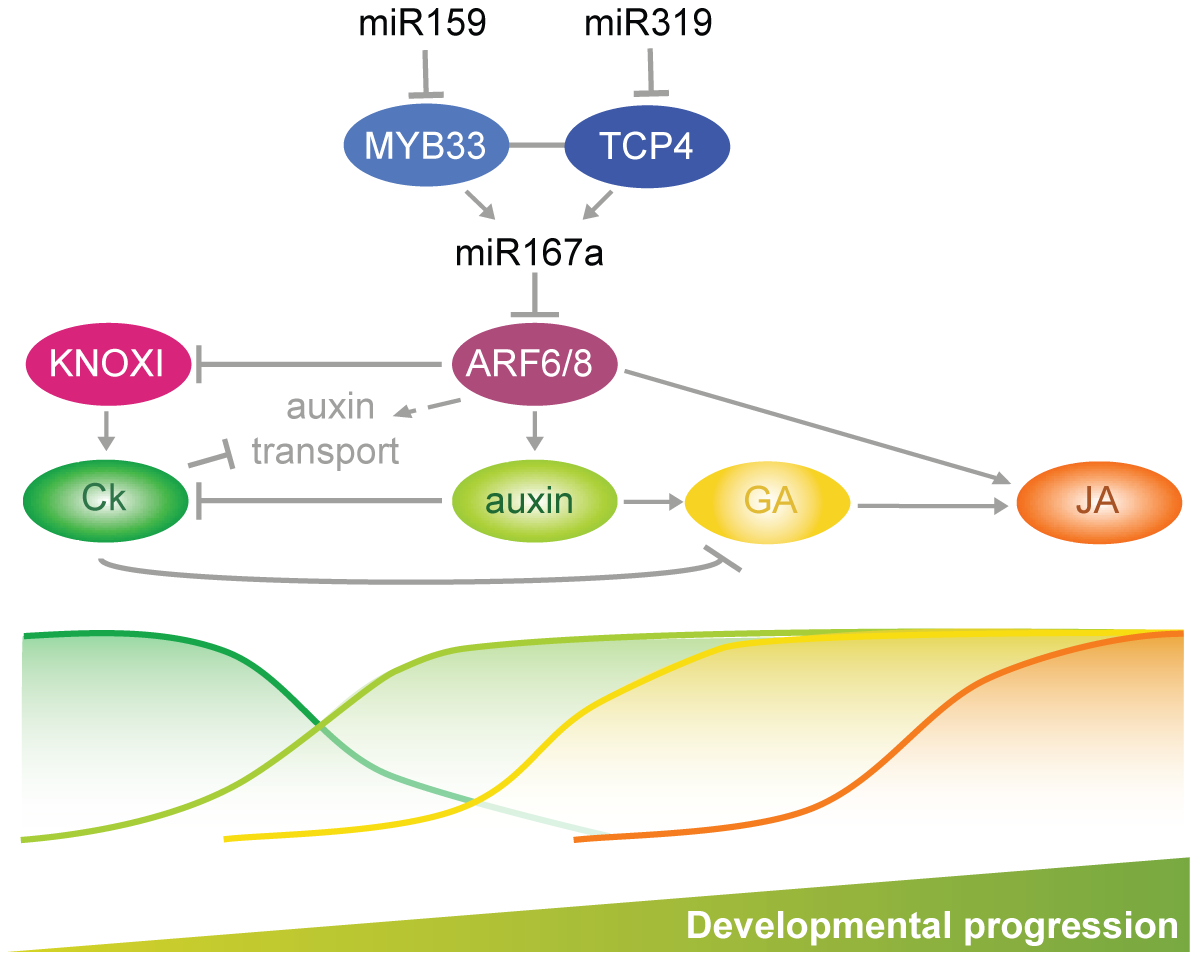
Summary of interactions described in this work driving developmental progression of petals and anthers.

Petal and anther development are particularly sensitive to perturbations in the miR159-miR167-miR319 network. Hormone action in the development of these floral organs starts with the maintenance of meristematic activity linked to *KNOXI*-dependent cytokinin biosynthesis and signal transduction (Figure 7). Auxin action is a prerequisite for the initiation of flower organ primordia [9], [49]–[51]. Later on, miR159- and miR319-dependent ARF6/8 activities control a checkpoint for a transition that requires inhibition of *KNOXI* genes, which in turn regulates auxin transport and GA signaling (Figure 7) [2], [7], [52], [53]. Both processes are essential for the elongation and maturation of petals and anthers as well as for JA biosynthesis, which contributes to the last steps of the maturation of both organs (Figure 7) [2], [4], [7], [8], [10], [11], [32], [33], [35], [54]. Auxin action, mediated by ARF5/MP and ARF6/8, also promotes cambium development [4], [55], [56], and we propose that progression of vascular development which appears to follow a similar sequence of signaling events as floral organ maturation (Figure 7) [36], [57], is mediated by the miR159-miR167-miR319 network as well.

Sepals, petals and stamens, which make up the three outer whorls of floral organs, have key roles in evolutionary transitions between different plant mating strategies. The most common change is from obligate cross-pollination (allogamy) to self-fertilization (autogamy), which is found in up to half of all flowering plants, including many domesticated species [58]. The window of time during which the stigma is exserted beyond the outcrossing-protective sepals and petals before the stamens elongate, is a central determinant of outcrossing opportunities [59], [60]. Since the miR159-167-miR319 network is crucial for the development of sepals, petals and stamens, modulation of its activity might provide a regulatory entry point for different reproductive strategies.

## Materials and Methods

### Plant material

Plants were grown on soil in long days (16 h light/8 hours dark) under a mixture of cool and warm white fluorescent light at 23°C and 65% humidity. *ProMIR167A:GUS*, *ProMIR167B:GUS*, *ProMIR167c:GUS ProARF8:ARF8-GUS*, *ProARF8:mARF8-GUS* and *afr6-*2 *arf8-*3 [1], [4], artificial miRNA target mimics [5], *ProLOX2:GUS* and *ProTCP4*:*TCP4-GFP* [31], the Q0990 marker [24], and *bp*-1 mutants [61] have been described.

### Transgenic plants

*MIR167A* and *MIR167C* fragments were PCR amplified from Col-0 genomic DNA, and placed behind the constitutive CaMV 35S promoter (Pro35S) [62]. Unmodified and modified *MYB33* and *TCP4* coding sequences [17] were placed behind the *AP3* promoter [26]. The *ProMIR319B:GUS* reporter included a 2,662 bp genomic DNA fragment that begins 182 bp upstream of the first nucleotide of pre-miR319b. The *MIR167A* promoter was according to [1]. See Table S2 for oligonucleotide primers. Constructs were introduced into Col-0 plants by *Agrobacterium tumefaciens*-mediated transformation [63]. Their names can be found in Table S3.

### RNA analyses

Total RNA was extracted from 30-day old inflorescences of T1 or F1 plants using TRIzol Reagent (Invitrogen) with two biological replicates using tissue pooled from 10 to 15 plants each. After reverse transcription with the RevertAid First Strand cDNA Synthesis Kit (Fermentas) of 1 µg of total RNA that had been treated with DNase I (Fermentas). PCR was carried out in presence of SYBR Green (Invitrogen) and monitored in real time with the Opticon Continuous Fluorescence Detection System (MJR/BioRad). For in situ hybridization [64] to detect *ARF6*/8, we used sections of inflorescences from 30-day old plants, with sense and antisense probes as described [1]. For LNA in situ hybridization [65] to detect miR167, 10% polyvinylalcohol was added to the colorimetric reaction buffer to increase the sensitivity of the assay.

### Histology

Inflorescences were fixed in 90% acetone, and GUS activity was assayed as described [66]. For *ARF8-GUS* reporters, ten-fold lower concentrations of ferri- and ferrocyanate were used to increase sensitivity. Gus pictures are representative of three different experiments that included at least 10 samples each. Petals were sequentially cleared with chloral hydrate and ethanol/glycerol/lactic acid (3∶1∶1).

### Chromatin immunoprecipitation

About 150 inflorescences were collected and fixed in two biological replicates, and immunoprecipitation was performed as described [67], with 3 µl of anti-rabbit GFP antibody (ab290; Abcam). Twenty µl eluate from Minelute columns (Qiagen) was diluted 1∶5 and used as template for two technical replicates of real-time PCR. Enrichment was calculated by comparing amplification in post-binding and input fraction and normalized to enrichment in samples from empty vector plants. We used a DNA fragment located within the 1 Kb upstream region of the *Hsf1* gene (at4g17750) and lacking any putative TCP binding site as negative control.

### Protein–protein interaction

Interaction in the yeast two-hybrid system was assayed on selective medium (Leu^−^, Trp^−^, His^−^) supplemented with 60 mM 3-amino 1,2,4-triazole (3-AT). The assay was repeated four times. For the luciferase complementation assay [69], fusions of non-targetable forms of MYB33, TCP2 and TCP4 were expressed from the 35S promoter. *Agrobacterium tumefaciens* cultures with the different constructs at OD_600_ = 0.3 each were mixed in equal ratios with a P19 silencing suppressor culture at OD_600_ = 0.1. Leaves were imaged three days after inoculation. Details for the constructs can be found in Table S3.

## Supporting Information

Figure S1 Expression levels of representative miR159 and miR319 targets in plants with specific miRNA attenuated function. (A) Expression of three representative miR319-TCP targets in *Pro35S:MIM319* inflorescences. (B) Expression of miR159-MYB targets in *Pro35S:MIM159* inflorescences. Expression was monitored by real-time RT-PCR. Error bars indicate range of two biological and two technical replicates. Expression values of the different genes in the mutant plants were normalized to their expression in wild type inflorescences (dashed lines).
(PDF)

Figure S2 Flowers and inflorescences of mutant and transgenic plants. (A) Scanning electron micrographs of flowers from transgenic plants. MIMXXX and MIRXXX lines express the target mimics and miRNAs from the 35S promoter. MIM159, MIM319 and MIR167C plants were 30 days, MIM167 plants 38 days old. Scale bar indicates 700 µm. (B) Entire inflorescences. Note that flowers grow more upwards, that is, at a smaller angle relative to the main stem, in MIM159 and MIM319 plants, or in *arf6 arf8* mutants. Scale bar indicates 2 mm.
(PDF)

Figure S3 MYB33 and TCP4 expression patterns in flowers. (A) *ProMYB33:GUS* is broadly express in every flower organ. (B) *ProTCP4:GUS* is expressed in the vasculature of sepals and anther filaments (procambium), petals and female reproductive organs.
(PDF)

Figure S4 Effect of miR159, miR167 and miR319 on *MIR167A* promoter activity, and comparison of *MIR167A* and *MIR319B* promoter activities. (A) Activity of *ProMIR167A:GUS* in different backgrounds. Note particularly strong ectopic activity in sepals, petals, and at the base of flowers and pedicels of MIM319 expressers. (B) Close-up of mature petals. Note ectopic activity in vasculature. (C) ProMIR319B:*GUS* expression in wild-type inflorescence. Activity is notable at the base of petioles and floral organs, and in anthers. Scale bars indicate 2 mm (A), 500 µm (B). See also Figure 5.
(PDF)

Figure S5 Regulation of *MIR167A* promoter by TCP transcription factors. (A) Reporter gene assay with *MIR167A* promoter. Mutations in either of the two TCP binding motifs suppress ectopic activity in response to MIM319 overexpression. Scale bar indicates 2 mm. (B) Heterodimerization of TCP2 and TCP4 assayed by firefly luciferase complementation assay in *N. benthamiana* leaves. Luciferase activity is shown in false color, with highest levels red and lowest levels blue. See also Figure 6.
(PDF)

Table S1 Potential R2R3MYB and TCP binding motifs in *MIR167A* and *LOX2* promoters.
(DOC)

Table S2 Oligonucleotide primer sequences.
(DOC)

Table S3 List of plasmids.
(DOC)
