# Supplementary figures and images for: Coordination of Flower Maturation by a Regulatory Circuit of Three MicroRNAs
Source: PLoS Genet. 2013 Mar 28;9(3):e1003374. doi: 10.1371/journal.pgen.1003374 (PMC3610633; doi:10.1371/journal.pgen.1003374)

**A**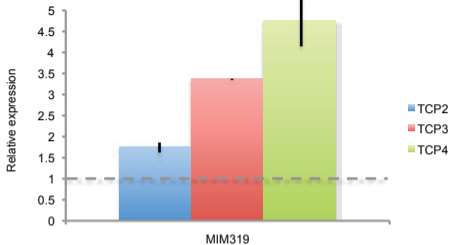**B**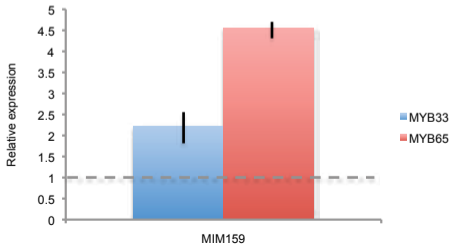

Supplement: Figure S1 — Expression levels of representative miR159 and miR319 targets in plants with specific miRNA attenuated function. (A) Expression of three representative miR319-TCP targets in Pro35S:MIM319 inflorescences. (B) Expression of miR159-MYB targets in Pro35S:MIM159 inflorescences. Expression was monitored by real-time RT-PCR. Error bars indicate range of two biological and two technical replicates. Expression values of the different genes in the mutant plants were normalized to their expression in wild type inflorescences (dashed lines). (PDF) [file pgen.1003374.s001.pdf]

**A**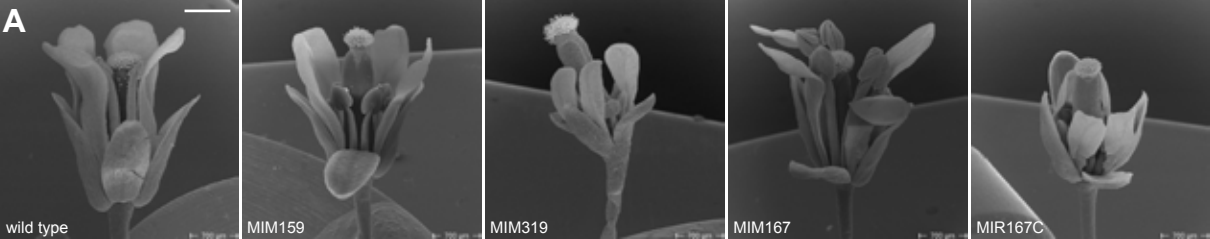**B**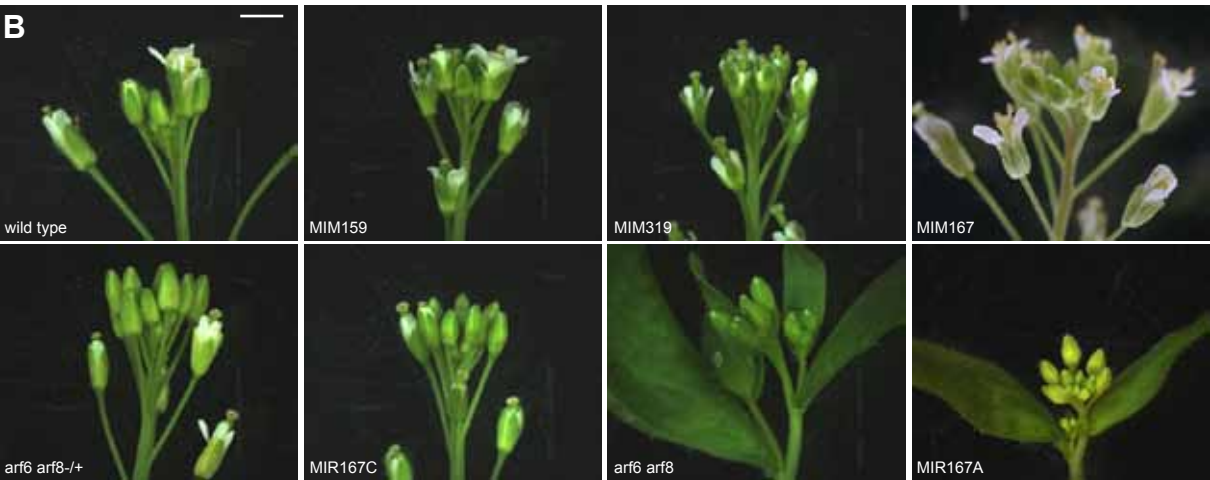

Supplement: Figure S2 — Flowers and inflorescences of mutant and transgenic plants. (A) Scanning electron micrographs of flowers from transgenic plants. MIMXXX and MIRXXX lines express the target mimics and miRNAs from the 35S promoter. MIM159, MIM319 and MIR167C plants were 30 days, MIM167 plants 38 days old. Scale bar indicates 700 µm. (B) Entire inflorescences. Note that flowers grow more upwards, that is, at a smaller angle relative to the main stem, in MIM159 and MIM319 plants, or in arf6 arf8 mutants. Scale bar indicates 2 mm. (PDF) [file pgen.1003374.s002.pdf]

**A**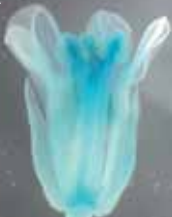

ProMYB33:GUS

**B**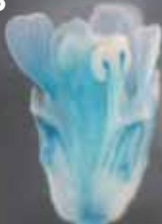

ProTCP4:GUS

Supplement: Figure S3 — MYB33 and TCP4 expression patterns in flowers. (A) ProMYB33:GUS is broadly express in every flower organ. (B) ProTCP4:GUS is expressed in the vasculature of sepals and anther filaments (procambium), petals and female reproductive organs. (PDF) [file pgen.1003374.s003.pdf]

**A**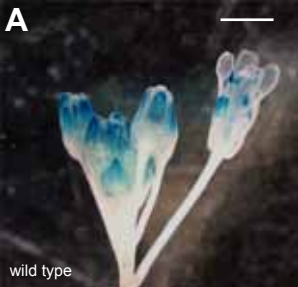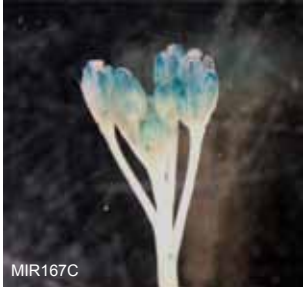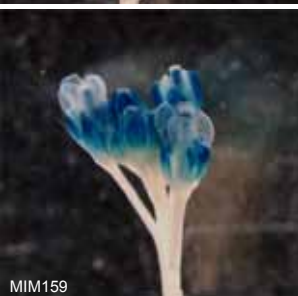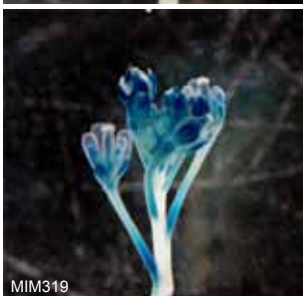**B**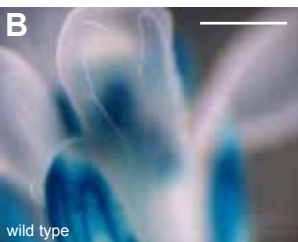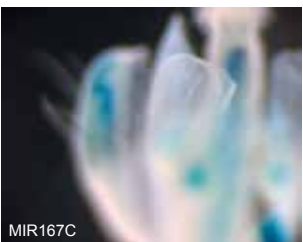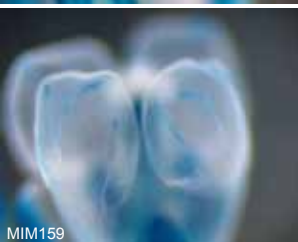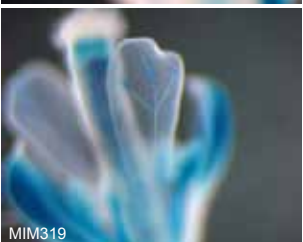**C**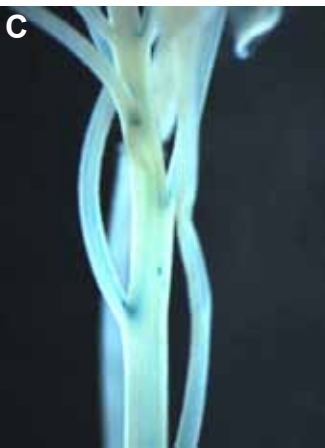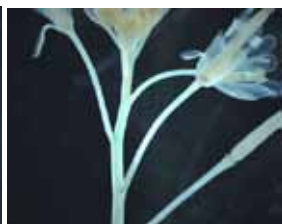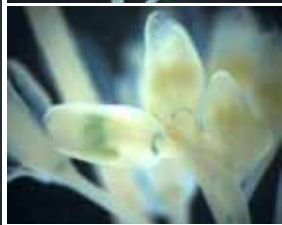

Supplement: Figure S4 — Effect of miR159, miR167 and miR319 on MIR167A promoter activity, and comparison of MIR167A and MIR319B promoter activities. (A) Activity of ProMIR167A:GUS in different backgrounds. Note particularly strong ectopic activity in sepals, petals, and at the base of flowers and pedicels of MIM319 expressers. (B) Close-up of mature petals. Note ectopic activity in vasculature. (C) ProMIR319B:GUS expression in wild-type inflorescence. Activity is notable at the base of petioles and floral organs, and in anthers. Scale bars indicate 2 mm (A), 500 µm (B). See also Figure 5. (PDF) [file pgen.1003374.s004.pdf]

**A**

mut 1

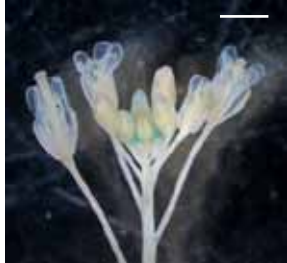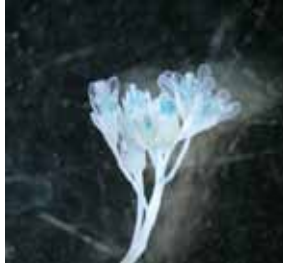

mut 2

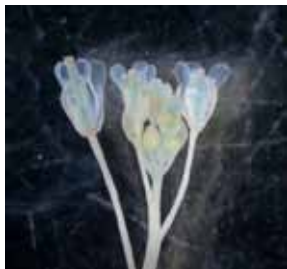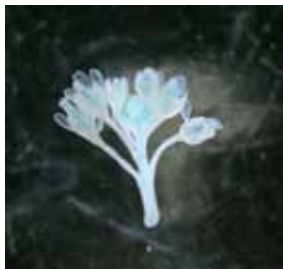

mut 1 mut 2

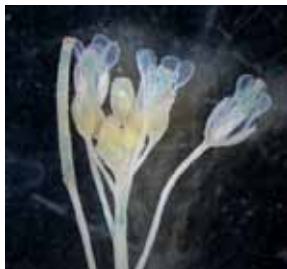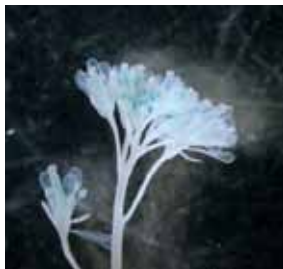

wild type

MIM319

**B**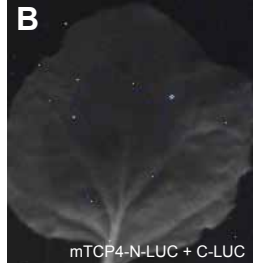

mTCP4-N-LUC + C-LUC

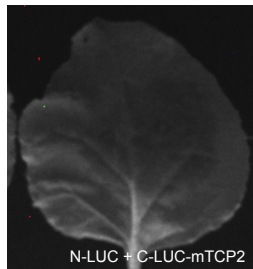

N-LUC + C-LUC-mTCP2

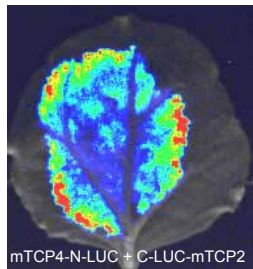

mTCP4-N-LUC + C-LUC-mTCP2

Supplement: Figure S5 — Regulation of MIR167A promoter by TCP transcription factors. (A) Reporter gene assay with MIR167A promoter. Mutations in either of the two TCP binding motifs suppress ectopic activity in response to MIM319 overexpression. Scale bar indicates 2 mm. (B) Heterodimerization of TCP2 and TCP4 assayed by firefly luciferase complementation assay in N. benthamiana leaves. Luciferase activity is shown in false color, with highest levels red and lowest levels blue. See also Figure 6. (PDF) [file pgen.1003374.s005.pdf]
