# Supplementary material for: Coordination of Flower Maturation by a Regulatory Circuit of Three MicroRNAs
Source: PLoS Genet. 2013 Mar 28;9(3):e1003374. doi: 10.1371/journal.pgen.1003374 (PMC3610633; doi:10.1371/journal.pgen.1003374)
Supplement: Table S1 — Potential R2R3MYB and TCP binding motifs in MIR167A and LOX2 promoters. (DOC) [file pgen.1003374.s006.doc]

**Table S1.** Potential R2R3-MYB and TCP Binding Motifs in *MIR167A* and *LOX2* Promoters.

| **Promoter** | **Factor** | **Site** | **Position^†^** | **Sequence** | **Reference*** |
| --- | --- | --- | --- | --- | --- |
| *MIR167A* | MYB | I | -1,898 to -1,894 | CAGTT | 1 |
| *MIR167A* | MYB | II | -1,645 to -1,640 | TTGTTA | 2 |
| *MIR167A* | MYB | III | -1,553 to -1,548 | CAGTT | 1 |
| *MIR167A* | MYB | IV | -1,198 to -1,193 | TTGTTA | 2 |
| *MIR167A* | MYB | V | -751 to -747 | CAGTT | 1 |
| *MIR167A* | MYB | VI | -622 to -617 | TAACAA | 2 |
| *MIR167A* | MYB | VII | -561 to -556 | TAACAA | 2 |
| *MIR167A* | TCP | 1 | -351 to -346 | GGTCC | 3 |
| *MIR167A* | TCP | 2 | -189 to -184 | GGACC | 3 |
| *LOX2* | TCP | 3 | -279 to -273 | GTGGTCC | 3 |

^†^relative to transcription start

*references:

1. Gocal, G.F. *et al.* Long-day up-regulation of a *GAMYB* gene during *Lolium temulentum* inflorescence formation. *Plant Physiol.* **119**, 1271-8 (1999).

2. Gubler, F., Kalla, R., Roberts, J.K. & Jacobsen, J.V. Gibberellin-regulated expression of a myb gene in barley aleurone cells: evidence for Myb transactivation of a high-pI alphaamylase gene promoter. *Plant Cell* **7**, 1879-91 (1995).

3. Schommer, C. *et al.* Control of jasmonate biosynthesis and senescence by miR319 targets. *PLoS Biol* **6**, e230 (2008).
