# Supplementary material for: Coordination of Flower Maturation by a Regulatory Circuit of Three MicroRNAs
Source: PLoS Genet. 2013 Mar 28;9(3):e1003374. doi: 10.1371/journal.pgen.1003374 (PMC3610633; doi:10.1371/journal.pgen.1003374)
Supplement: Table S2 — Oligonucleotide primer sequences. (DOC) [file pgen.1003374.s007.doc]

**Table S2.** Oligonucleotide Primer Sequences.

| Primer name | Purpose | Sequence |
| --- | --- | --- |
| miR167a.s | Overexpression | GGATCCCTTCTTCACTTGATGAACAG |
| miR167a.as | Overexpression | GTCGACCAGCATATCTGTGGTTAATC |
| miR167c.s | Overexpression | CCTGAATTCCTTGTAACTTAAGATATCC |
| miR167c.as | Overexpression | CCTGTCGACCATGATTGTCACACTAGCAC |
| Plox2.s | ChIP | AATGGCCGGTTCCTCCAAAACTAG |
| Plox2.as | ChIP | CTTACCCGATATTGCATGTGATGC |
| HSF1.s | ChIP | TATAGCAACCTCTGAGCCTCTTG |
| HSF1.as | ChIP | GAGAAAGATTGTGTGAGAATGAAA |
| PmiR167aA.s | ChIP | CTTTATTTCCAAAAACCAAGAATAAGAAG |
| PmiR167aA.as | ChIP | GCCTCATGCACTTCTCTAATCTC |
| PmiR167aB.s | ChIP | GAGAAGTGCATGAGGCTCGGAGAATCG |
| PmiR167aB as | ChIP | GGAGAGTTTAAGGGTCGAAAGTG |
| PmiR319b.s | GUS reporter | GTACTTGTTGAAGTTCAATGTAC |
| PmiR319b.as | GUS reporter | GCGTGCTAGAATCAATTCTAATC |
| PmiR167a.s | GUS reporter | GTTTCGAGTAGACCGTGAC |
| PmiR167a.as | GUS reporter | GTTTATATAGAAGGGATAAGG |
| PmiR167am1.s | Promoter mutagenesis | GAAAAAAAGAGGTTCCCCATCAATGTAG |
| PmiR167am1.as | Promoter mutagenesis | CTACATTGATGGGGAACCTCTTTTTTTC |
| PmiR167am2.s | Promoter mutagenesis | CACGGTTCTACTTTGGAACACTTTTCTTTAACGCC |
| PmiR167am2.as | Promoter mutagenesis | GGCGTTAAAGAAAAGTGTTCCAAAGTAGAACCGTG |
| ProMYB33.s | GUS reporter | CCCcaattgCGATCAAGTCAAGAAGGTCGTC |
| ProMYB33.as | GUS reporter | CCCggtaccctcttttctaattaaaccactgac |
| ProTCP4.s | GUS reporter | ATGCcAATtGTGACCGTAATTCCAAGTGGAAG |
| ProTCP4.as | GUS reporter | cgcggtaccCTCTCTCTATTGTTTGGTCTGTGT |
| QRTPIN1.s | qRT-PCR | GGAGACTTAAGTAGGAGCTCAGCA |
| QRTPIN1.as | qRT-PCR | CCAAAAGAGGAAACACGAATG |
| QRTSTM.s | qRT-PCR | GGATAATAGTGATGGTCCGA |
| QRTSTM.as | qRT-PCR | GGGGAGGAGCTAGTGATTGA |
| QRTBP.s | qRT-PCR | GAGAATTGCTTCCGATCT |
| QRTBP.as | qRT-PCR | ATGGCTTCAACATCGCTTAC |
| QRTMYB33.s | qRT-PCR | TCCCTTCATTCCAATATTCAG |
| QRTMYB33.as | qRT-PCR | GAGTTTCATCTGCATTTTGTGTG |
| QRTMYB65.s | qRT-PCR | ACGTATGGCATGCATCCTACTTCTAAG |
| QRTMYB65.as | qRT-PCR | TAACATTGGTCGTGCAAGTAGTTGAAC |
| QRTTCP2.s | qRT-PCR | ATATCACCGGCAGAATCCAA |
| QRTTCP2.as | qRT-PCR | TGTGGACCTCCTCCACTTTC |
| QRTTCP3.s | qRT-PCR | CGGAGGATTTGTGTTTGCTT |
| QRTTCP3.as | qRT-PCR | AGGGATGATGATGGTGGAGA |
| QRTTCP4.s | qRT-PCR | CCTTCAACGACGTCGTTTCAGCCAG |
| QRTTCP4.as | qRT-PCR | GTGAACCGGTGGAGGAAGGTGATG |
| QRTARF6.s | qRT-PCR | GCGAAGCGAGCTTGCTCG |
| QRTARF6.as | qRT-PCR | CATTTGTTGCACTTCTTGTGG |
| QRTARF8.s | qRT-PCR | TTCAGCAGCTACCACGAGCTG |
| QRTARF8.as | qRT-PCR | CCACCACTGCCTTCTCCATG |
| MYB33.s | Bimolecular luminiscence complementation | TTGGATCCATGAGTTACACGAGCAC |
| MYB33.as | Bimolecular luminiscence complementation | TTGTCGACTTAGGGTAGTTCTGTC |
| TCP2.s | Bimolecular luminiscence complementation | TTGGATCCATGATTGGAGATCTAATG |
| TCP2.as | Bimolecular luminiscence complementation | TTGTCGACGTTCTTGcCTTTACCCTTATG |
