# Supplementary material for: Coordination of Flower Maturation by a Regulatory Circuit of Three MicroRNAs
Source: PLoS Genet. 2013 Mar 28;9(3):e1003374. doi: 10.1371/journal.pgen.1003374 (PMC3610633; doi:10.1371/journal.pgen.1003374)
Supplement: Table S3 — List of plasmids. (DOC) [file pgen.1003374.s008.doc]

**Table S3.** List of Plasmids.

| Construct | ID | Description |
| --- | --- | --- |
| Pro35S:pre-MIR167A | pIR156 | Pre-MIR167A overexpression |
| Pro35S:pre-MIR167C | pIR159 | Pre-MIR167C overexpression |
| ProAP3:MYB33 | pIR181 | MYB33 expression in APETALA3 domain |
| ProAP3:mMYB33 | pIR183 | mMYB33 expression in APETALA3 domain |
| ProAP3:TCP4 | pIR185 | TCP4 expression in APETALA3 domain |
| ProAP3:mTCP4 | pIR187 | mTCP4 expression in APETALA3 domain |
| BD-mMYB33 | pIR189 | mMYB33 fusion to DNA-binding domain in pDEST32 for Y2HS |
| AD-mTCP4 | pIR190 | mMYB33 fusion to Activation domain in pDEST22 for Y2HS |
| BD-mTCP2 | pIR192 | mTCP2 fusion to DNA-binding domain in pDEST32 for Y2HS |
| 35S:N-LUC | pJW771 | Bimolecular luminescence complementation assays |
| 35S:C-LUC | pJW772 | Bimolecular luminescence complementation assays |
| 35S:C-LUC-mMYB33 | pIR193 | Bimolecular luminescence complementation assays |
| 35S:C-LUC-mTCP2 | pIR194 | Bimolecular luminescence complementation assays |
| 35S:mTCP4-N-LUC | pJW790 | Bimolecular luminescence complementation assays |
| ProMIR167AmTCP1:GUS | pIR163 | MIR167A promoter fusion to GUS reporter gene mutated in TCP binding site 1 |
| ProMIR167AmTCP2:GUS | pIR165 | MIR167A promoter fusion to GUS reporter gene mutated in TCP binding site 1 |
| ProMIR167AmTCP1,2:GUS | pIR167 | MIR167A promoter fusion to GUS reporter gene mutated in TCP binding site 1 and 2 |
| ProMIR319B:GUS | pIR169 | MIR319B promoter fusion to GUS reporter gene |
| ProMYB33:GUS | CS50 | MYB33 promoter fusion to GUS reporter gene |
| ProTCP4:GUS | CS36 | TCP4 promoter fusion to GUS reporter gene |
| ProAP3:MIM159 | pIR81 | MIM159 expression in APETALA3 domain |
| ProAP3:MIM319 | pIR95 | MIM319 expression in APETALA3 domain |
